# Osteochondral Fracture Lateral Femoral Condyle Treated with ORIF Using Z-Plasty: A Modification of Coonse and Adams Approach

**DOI:** 10.1155/2011/957196

**Published:** 2011-10-19

**Authors:** Sanjay Agarwala, Ganesh S. Mohrir, Brijbhushan S. Mahajan

**Affiliations:** Department of Orthopaedics, P. D. Hinduja National Hospital and Medical Research Centre, Mahim, Mumbai 400016, India

## Abstract

Osteochondral fractures of lateral femoral condyle are common in adolescents and young adults. They are usually caused by direct trauma or twisting injuries of the knee. We present a case of large osteochondral fracture of lateral femoral condyle involving the articular surface in a fifteen-year-old male with a positive history of significant weight gain of 5 kilograms in last six months. Blood investigations reported low vitamin D and testosterone levels with elevated alkaline phosphatase. Adequate exposure was achieved by doing Z-plasty of quadriceps apparatus. The fracture was treated with open reduction and internal fixation using Herbert's screws. Medical management in the form of vitamin D and calcium along with testosterone was given. After the surgery, full weight-bearing was allowed at three months. At one year followup, patient has good quadriceps function without any weakness of the muscle.

## 1. Case Report

**Figure 1:**
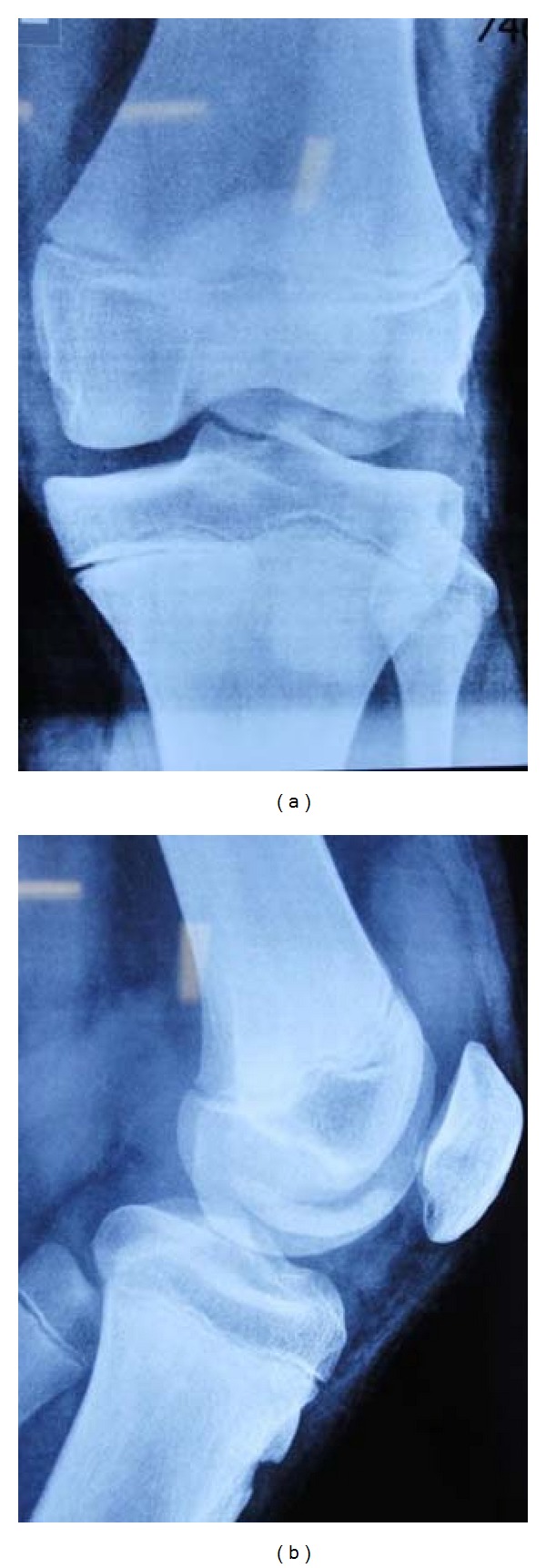
Radiographs of knee joint, (a) anteroposterior and (b) lateral.

**Figure 2:**
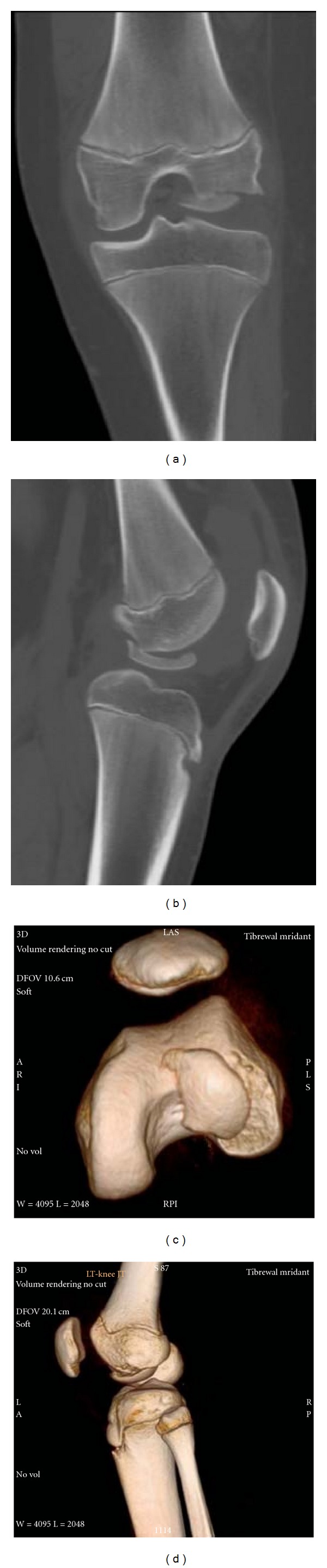
CT scan with 3D reconstruction.

A fifteen-year-old boy sustained a trivial injury to his left knee while turning on weight-bearing knee joint. He presented with severe pain and a swelling around knee joint and inability to bear weight on injured limb. On examination, flexed attitude of knee joint was evident. Tenderness over lateral femoral condyle was detected with restricted movements. No instability was detected. All ligament tests were normal. Routine AP and lateral radiographs showed large osteochondral fracture of lateral femoral condyle (Figures 1(a) and 1(b)). CT scan with 3D reconstruction was done to understand the anatomy of the fracture and to rule out any associated injuries (Figures 2(a), 2(b), 2(c), and 2(d)).

The patient was investigated as the force of injury was inadequate to cause a traumatic fracture. The serum vitamin D levels were 13.8 ng/mL (normal = above 80 ng/mL) and serum testosterone level of 0.93 ng/mL along with alkaline phosphatase levels increased to 187 from normal range of 40–120. Hence we felt this was a pathological fracture.

**Figure 3:**
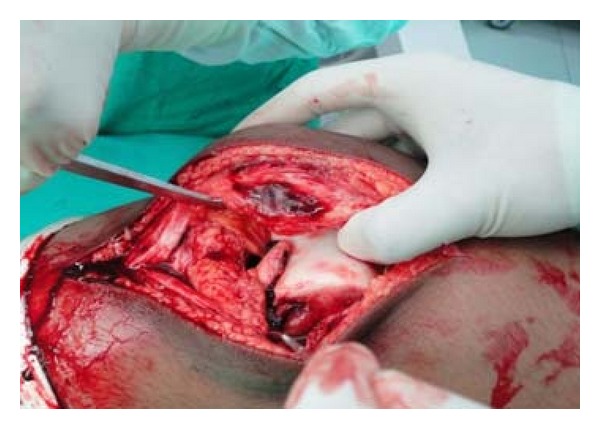
Photograph showing inadequacy of exposure.

 We decided on open reduction and internal fixation of the fracture as with arthroscopy introducing Herbert's screws perpendicular to fracture site was not possible. An anterior midline skin was taken. Skin flap was elevated mainly on lateral side to aid exposure. A lateral parapatellar arthrotomy was done. There was inadequate exposure with this approach (Figure 3). Though the anterior aspect of the fractures fragment was clearly visible, posterior part of the fractured fragment and its bed on femoral condyle were not visible enough to perform anatomical reduction, neither in flexion nor extension. The space obtained by this exposure was not enough to accommodate instrumentation to put Herbert's screws in direction perpendicular to fracture direction. We discussed on table the option of excision of infrapatellar fat pad and came to conclusion that it would expose the anterior part of fracture more clearly but visualisation of posterior aspect would still be inadequate.

**Figure 4:**
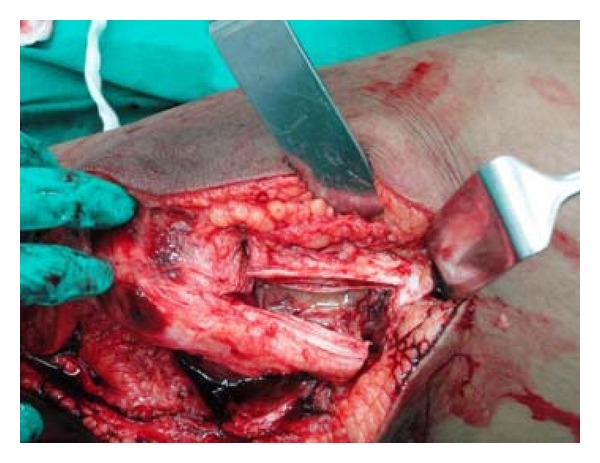
Approach with Z plasty (Modified Coonse and Adams approach).

**Figure 5:**
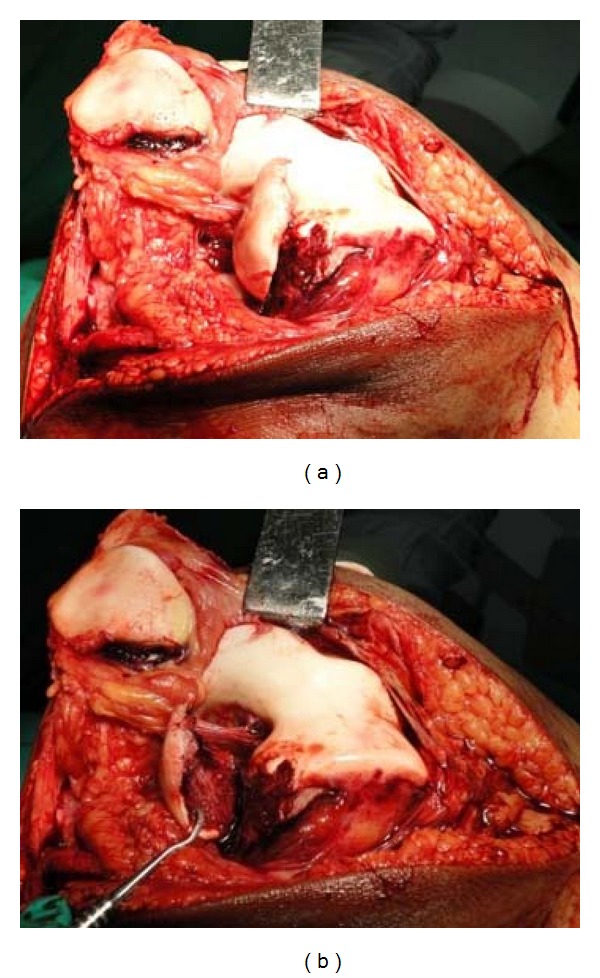
Exposure before Z-plasty (a) and after Z-plasty (b).

**Figure 6:**
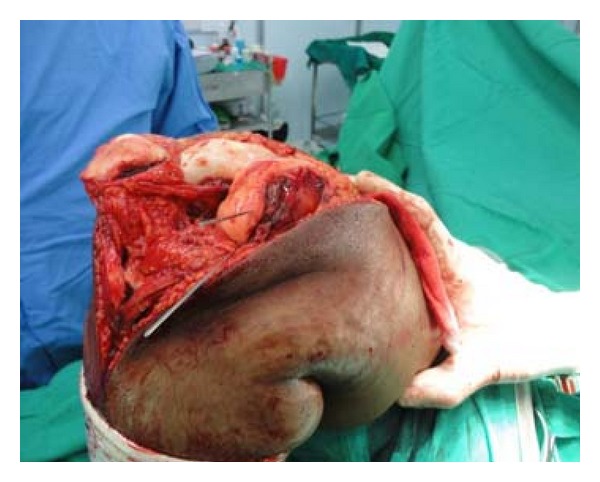
Temporary k wire fixation.

**Figure 7:**
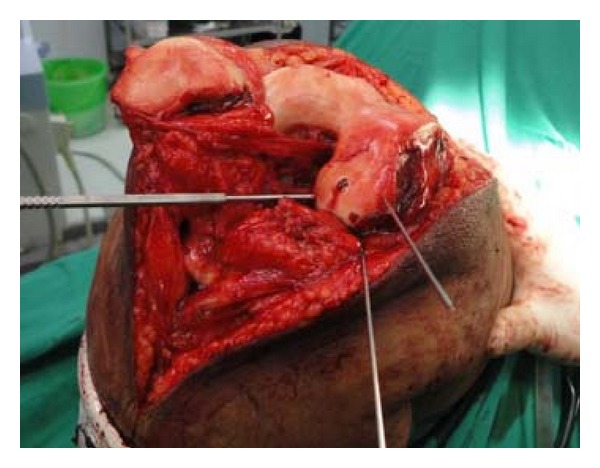
Herbert's screw fixation.

**Figure 8:**
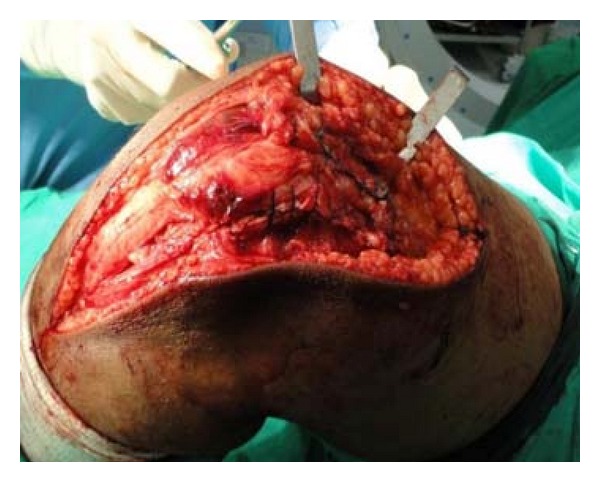
Secure closure of quadriceps.

Hence, to obtain good exposure for anatomical reduction and rigid fixation as well as to get space for use of instrument of definite fixation, Z-plasty of quadriceps apparatus was done (Figure 4, modification of Coonse and Adam's approach). With Z-plasty, posterior part of lateral femoral condyle was seen clearly (Figure 5(a)) and fracture bed was nicely visible (Figure 5(b)). Anatomical reduction of fracture was done and fracture was fixed temporarily with 1.2 mm K-wires (Figure 6). Definite and stable fixation with Herbert's screws was done (Figure 7) following the AO principles for intra-articular fractures. Postfixation quadriceps was securely sutured (Figure 8). 

**Figure 9:**
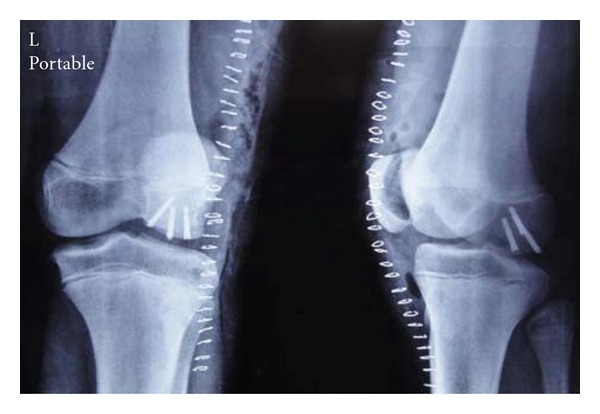
Postoperative radiograph.

**Figure 10:**
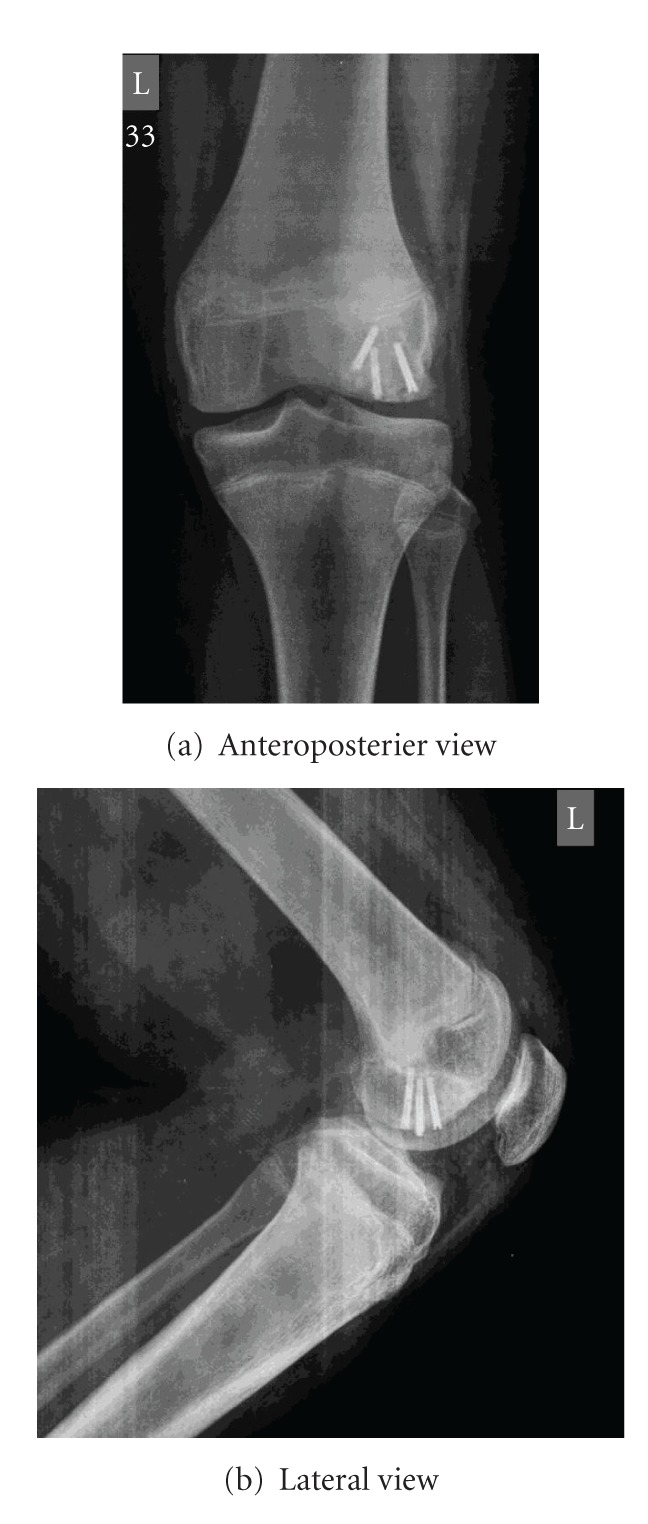
Radiograph at one year follow-up.

Immediate postoperative radiographs were done. Good anatomic reduction with stable fixation achieved (Figure 9). The patient was immediately mobilised in bed as quadriceps apparatus was securely fixed. Patient was encouraged to do active movements of the knee joint and was advised nonweight bearing for three months postoperatively. At one year follow up patient managing all activities including sports without any deficiency or weakness of quadricep muscles. Radiographs shows good healing without any articular cartilage or subchondral bone changes (Figures 10(a) and 10(b)).

## 2. Discussion

Large osteochondral fractures are uncommon. Anteroposterior, lateral, skyline, tunnel, and oblique radiographs should be obtained if an osteochondral fracture is suspected as early diagnosis is essential for primary fixation [1, 2]. Undisplaced osteochondral fractures in children often can be treated successfully with conservative methods. Displaced osteochondral fractures should be treated operatively. Surgery should be performed promptly to prevent further damage to the joint. Frequently, fragments are so small that fixing them is impossible; these should be excised. Mathewson and Dandy [3] recommended early primary repair of large osteochondral fractures as the articular surface begins to fill with fibrocartilage within ten days of injury.

Usual approach to knee joint exposure is anterior midline skin incision and lateral or medial parapatellar arthrotomy. Though when the fracture involves majority of posterior articular surface, exposure with such an approach is very limited and space for using any fixation technique is inadequate. 

Various approaches have been described for getting good exposure. Coonse and Adams originally described patella turndown approach where they used paramedian skin incision. Arthrotomy was done by making inverted V-shaped incision with apex, 1 cm above the upper pole of patella. Soon they modified the skin incision to anterior midline incision and medial parapatellar arthrotomy. Second incision made at 45° from apex of quadriceps tendon extending to lateral side [4]. 

Insall modified the technique by making a quadriceps snip [5]. Another technique describes osteotomy of tibial tubercle and reflecting the whole quadriceps apparatus [6, 7]. However, with all these techniques, weakness of quadriceps was very common. Tibial tubercle nonunion was also reported with tibial tubercle osteotomy technique [6]. 

We used a different technique by doing Z plasty of quadriceps tendon extending the lateral parapatellar arthrotomy incision. The patient had grade five power of quadriceps at follow-up visits and did not report any weakness of muscle power.

MRI or CT scan are noninvasive and can show these fractures very well, but can only be used for diagnosis. CT is helpful in determining the size and location of the fragment and its site of origin. The aim of primary fixation is to restore the joint surface and prevent secondary osteoarthritis. Fixation of large osteochondral fractures can be achieved with metal pins, allograft cortical bone pins, Herbert screws, absorbable sutures, and biodegradable screws [8, 9]. The potential but rare long-term effects of using biodegradable screws are synovitis, osteosclerosis, aseptic swelling, and osteolytic radiographic changes. The defect is bony as well as cartilaginous and therefore cartilage “resurfacing” or marrow stimulation techniques such as microfracture, drilling, and autologous chondrocyte implantations [10] are not likely to be successful. The long-term outcome of mosaicplasty for a large osteochondral lesion is unpredictable [11], and this technique has donor site morbidity.

## 3. Conclusion

Z-plasty of quadriceps apparatus is effective alternate technique to obtain adequate exposure and hence allows anatomical reduction of fracture and provides wide area of exposure for fixation. 

With this technique, good quadriceps function can be achieved in the postoperative period without any weakness of quadriceps muscle. Early fixation of these injuries is likely to give the best results.
